# Effect of Substrate Compliance on the Jumping Mechanism of the Tree Frog (*Polypedates dennys*)

**DOI:** 10.3390/biomimetics10090604

**Published:** 2025-09-09

**Authors:** Rui Zhou, Baowen Zhang, Zhouyi Wang, Zhendong Dai

**Affiliations:** 1 College of Mechanical and Electrical Engineering, Nanjing University of Aeronautics and Astronautics, Nanjing 210016, China; zhourui@nuaa.edu.cn (R.Z.); baowen.zhang@wur.nl (B.Z.); zddai@nuaa.edu.cn (Z.D.); 2 Experimental Zoology Group, Wageningen University & Research, 6708 WD Wageningen, The Netherlands; 3 Shenzhen Research Institute, Nanjing University of Aeronautics and Astronautics, Shenzhen 518063, China

**Keywords:** compliant substrate, jumping mechanism, tree frog, energy recovery, kinematics

## Abstract

Animal locomotion in complex environments depends on the ability to adaptively regulate movement in response to substrate mechanics. Tree frogs (*Polypedates dennysi*), which combine jumping and adhesive capabilities, inhabit arboreal habitats with a wide range of compliant substrates. While previous studies have offered preliminary insights into their locomotion, the biomechanical mechanisms underlying their adaptability remain poorly characterized. In this study, we developed a stiffness-adjustable takeoff substrate supported by four springs, and combined it with a 3D motion capture system to analyze the jumping dynamics and kinematics of frogs across a broader range of compliant substrates. We found that energy recovery from the substrate was influenced by compliance. On the stiffest substrate, up to 50% of the stored energy was recovered during takeoff, whereas highly compliant substrates caused nonlinear damping, energy dissipation, and even takeoff failure. During takeoff, frogs generated peak normal forces up to 6 times their body weight and fore–aft forces up to 4.5 times their body weight. However, force generation showed limited adaptability to substrate mechanics, while takeoff velocity exhibited stronger adaptability to changes in compliance. These findings reveal a trade-off between substrate mechanics and jump performance. This work provides biomechanical insight into substrate preference and informs the design of bioinspired systems capable of efficient locomotion on compliant substrates.

## 1. Introduction

The behavioral and morphological adaptations of species to their physical environment play a crucial role in their survival and reproduction. In terms of locomotion, numerous species have evolved movement patterns and morphological characteristics that are adapted to their environments over long evolutionary timescales [1,2,3,4]. For instance, sand lizards (*Uma scoparia* and *Callisaurus draconoides*) have evolved elongated feet and specialized toe fringes to enhance mobility on loose, granular substrates [5]. Water striders exploit superhydrophobic structures formed by nanoscale setae on their legs to harness surface tension, enabling rapid gliding and jumping on the water surface [6]. Basilisk lizards utilize a combination of unique foot morphology and rapid slapping motions to generate stable air pockets beneath their feet, allowing them to run across water surfaces, a phenomenon often referred to as “water walking” [7,8]. Geckos, on the other hand, achieve reliable adhesion even on violently oscillating leaves by means of van der Waals forces generated through dense arrays of nanoscale setae on their toe pads [9]. These examples illustrate how organisms develop morphological and behavioral adaptations to meet locomotor challenges posed by substrate mechanical properties. Such adaptive strategies are especially critical when animals interact with dynamic substrates, including those that are elastic or whose effective stiffness changes during locomotion (e.g., through variations in substrate diameter or unpredictable energy return [10,11,12]). In these contexts, effective locomotion requires animals to synchronize their mechanical output with the phase-specific feedback of the substrate in order to achieve efficient propulsion.

Among various locomotor modes, jumping is particularly sensitive to changes in substrate mechanical properties due to its requirement for high instantaneous power output [13]. This sensitivity is especially evident when the takeoff substrate compliance increases. For example, in click beetles (Elateridae), jump height decreases significantly on more compliant substrates, and a 50% reduction in substrate compliance can significantly limit their ability to jump, leaving them capable only of a weak flipping motion [14]. Similarly, in the diamond dove (*Geopelia cuneata*), takeoff velocity is substantially reduced on compliant perches, which explains their natural preference for stiffer substrates in the wild [15]. In lizards (*Anolis carolinensis*), jumping performance is correlated with substrate compliance, with both takeoff velocity and jump distance declining significantly as perch compliance increases [16]. Mammals also exhibit such sensitivity: tree squirrels display divergent mechanical strategies according to the type of substrate, prioritizing force production on flat ground versus center of mass (COM) displacement on narrower poles. This adjustment is likely related to the coupling of substrate diameter and compliance in natural supports, where compliant substrates may compromise jumping performance by absorbing mechanical work [17]. Adult migratory locusts (*Locusta migratoria*) exhibit greater adaptability, they optimize their takeoff strategy by adjusting the timing of leg muscle activation and modifying the angle of leg extension to match substrate resonance frequencies, thereby not only adjusting their jumping kinematics in response to substrate mechanics but also partially recovering energy from substrate deformation [18]. These interspecific differences highlight the diverse adaptive strategies that have evolved in response to varying substrate compliance conditions.

**Figure 1:**
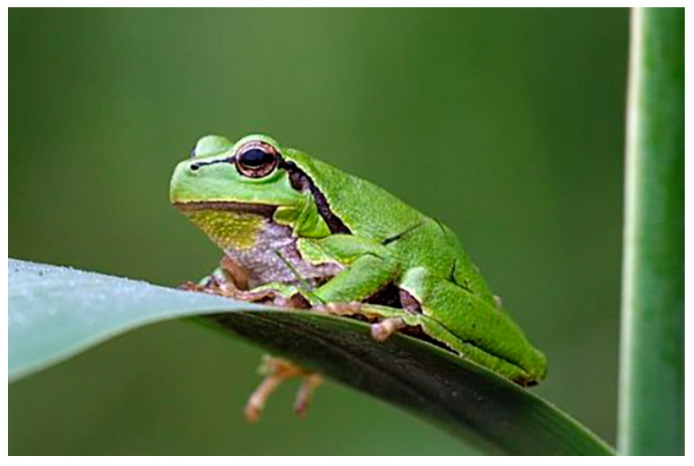
Tree frog locomotion on compliant substrates, including foliage. (Image source: www.quanjing.com, accessed on 20 June 2021).

Tree frogs, as highly specialized three-dimensional jumpers and exceptional adhesive climbers [19,20,21,22,23,24], inhabit a wide range of plant based substrates that vary from soft leaves (Figure 1) to stiff branches. These substrates differ by orders of magnitude in terms of compliance, surface curvature [20], and dynamic stability. Such complex environments present unique locomotor challenges for tree frogs. They must adapt to rapid changes in substrate compliance, achieve precise landings on vertical or even inverted surfaces [25], and cope with humidity-induced variations in adhesive performance [26]. Although previous studies have indicated that tree frogs can recover substrate energy during takeoff by modulating limb extension [27,28], these studies have primarily focused on behavioral observations, lacking direct mechanical measurements, and have been constrained by a narrow range of substrate compliance. These limitations have restricted a deeper understanding of the underlying mechanisms of takeoff on compliant substrates. To address this gap, we developed a takeoff platform with a broader adjustable stiffness range, integrated with synchronized motion capture, to quantify substrate deformation, elastic energy storage, and associated kinematic and kinetic variables. Specifically, we sought to determine whether tree frogs are still able to recover energy on more compliant substrates and whether peak takeoff forces decrease as substrate compliance increases. This experimental framework provides new insights into the biomechanical strategies underlying adhesive locomotion on compliant substrates and carries important implications for the design of bioinspired climbing and jumping robots.

## 2. Materials and Methods

### 2.1. Performance Testing Apparatus

Six active individuals of the large tree frog (*Polypedates dennysi*) were obtained from commercial suppliers and used in this study. Their average body mass was 71.9 ± 10.6 g and a snout–vent length (SVL) of 9.2 ± 0.5 cm. Each frog was housed individually in a glass enclosure measuring 0.4 m (length) × 0.4 m (width) × 0.5 m (height), equipped with a water source. The animals were fed three times per week with *Gryllus bimaculatus*. The husbandry conditions were maintained at a temperature of 26–29 °C, with a 12-h light/dark cycle and relative humidity ranging from 70% to 90%.

All research on animals was performed under the guidelines of the Chinese Regulations for the Management of Laboratory Animals. The laboratory has obtained long-term ethical approval for animal-related experiments from the Jiangsu Provincial Society for Laboratory Animals Science (Approval No. 2010012, Date: 2010), which remained valid during the conduct of this study. Specifically, the experiments in this study were carried out from July to September 2022, and all operations strictly adhered to the latest ethical regulations and the requirements specified in the above approval.

### 2.2. Experiment Setup

**Figure 2:**
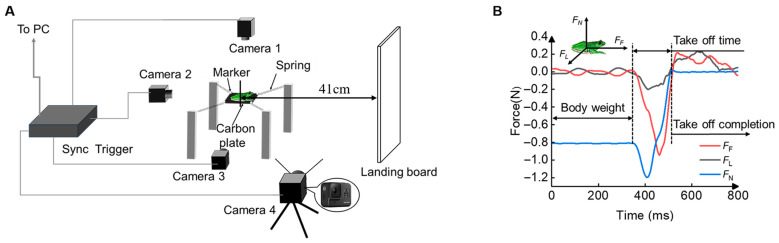
Measurement of jumping performance in tree frogs from substrates of various compliance. (**A**) Diagram of the experimental setup showing the location of the jump substrate and cameras used to record takeoff behavior. (**B**) A typical example of substrate reaction force, calculated using the force measurement system, during takeoff is shown, where *F*_F_ represents the fore–aft force, *F*_L_ denotes the lateral force, and *F*_N_ indicates the normal force. The initial normal force corresponds to the body weight of the tree frog, and after takeoff, the compliant substrate oscillates around zero.

Figure 2A illustrates the experimental setup employed in this study. The system consists of three main modules: a variable-stiffness takeoff substrate, a multi-camera motion capture system, and a synchronized data acquisition and processing unit. The variable-stiffness substrate is composed of a 12 cm × 13.6 cm carbon fiber plate constrained by four springs of different stiffness values. By adjusting the stiffness of individual springs, the substrate can simulate substrates of varying compliance. In the initial state, the substrate remains in a mechanically balanced state without external forces. When a tree frog initiates a jump from the substrate, the forces applied by its hindlimb cause displacement of the substrate. Owing to the linear mechanical properties of the springs, namely, that force is proportional to deformation, the force exerted on each spring can be calculated by tracking the spatial displacement of its two endpoints in real time. Since the substrate is only subject to the frog’s forces and the reactive forces from the springs, the net reaction force from all four springs corresponds to the ground reaction force generated by the frog’s feet. Based on this mechanical principle, the system enables real time quantification of the interaction forces between the frog’s feet and the compliant substrate during takeoff. Let *K* denote the stiffness coefficient of the springs, and let *A*_1_, *A*_2_, *A*_3_, and *A*_4_ represent the spatial coordinates of the fixed ends of the four springs, which are attached to the base frame, while the other ends are connected to the corners of the compliant substrate. The corresponding coordinates of the movable marker points located at the four corners of the compliant substrate are denoted as *M*_1_, *M*_2_, *M*_3_, and *M*_4_. The force vectors for the four springs are then given by:

(1) $$\left\{\begin{array}{l} \vec{F_{1}}=K(A_{1}-M_{1})\\ \vec{F_{2}}=K(A_{2}-M_{2})\\ \vec{F_{3}}=K(A_{3}-M_{3})\\ \vec{F_{4}}=K(A_{4}-M_{4}) \end{array}\right.$$

Therefore, substrate reaction force can be defined as:

(2) $$F=\vec{F_{1}}+\vec{F_{2}}+\vec{F_{3}}+\vec{F_{4}}-G$$

Here, *G* denotes the gravitational force vector generated by the weight of the substrate, which acts vertically downward and can be determined in advance. This approach enables not only the measurement of the resultant substrate reaction force exerted by the frog’s feet, but also the real-time determination of its direction.

In this study, to systematically modulate the stiffness of the takeoff substrate, springs with varying specifications were selected to achieve the target stiffness coefficients. We initially selected six types of commercially available springs, whose wire diameter multiplied by outer diameter yielded the following combinations: 0.7 mm × 3 mm, 0.6 mm × 3 mm, 0.5 mm × 3 mm, 0.5 mm × 4 mm, 0.3 mm × 5 mm, and 0.3 mm × 2 mm, all maintaining a consistent length of 27 mm. These springs were arranged in descending order of stiffness, with calculated stiffness coefficients of 6.34 N/cm, 2.58 N/cm, 0.87 N/cm, 0.32 N/cm, 0.11 N/cm, and 0.03 N/cm, respectively, demonstrating an approximately threefold difference between adjacent groups. In our preliminary experiments, we observed a critical threshold: when spring stiffness was ≤0.11 N/cm, tree frogs were unable to execute successful jumps. Although the frogs initiated takeoff movements, they consistently failed to detach their toe pads from the substrate. This phenomenon resulted from excessive substrate compliance, which prevented the generation of sufficient propulsive force for complete detachment. Therefore, although all six spring configurations were initially tested, only those with stiffness coefficients ≥0.32 N/cm were included in the final experimental protocol to ensure reliable jump performance. The overall stiffness of the takeoff substrate was then calculated accordingly, based on the combined stiffness of the selected springs.

**Figure 3:**
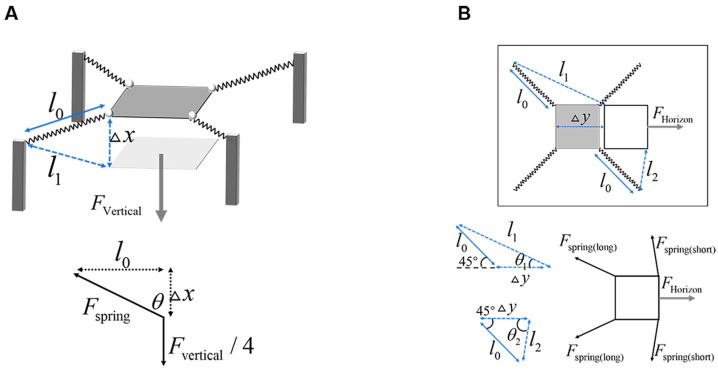
Stiffness calculation of the compliant takeoff substrate. (**A**) Illustrates the deformation of the substrate under vertical loading. The relationship between the deformed spring length and the vertical displacement can be derived based on geometric principles, allowing the calculation of spring force as a function of vertical displacement. Force analysis yields the relationship between the applied vertical load and the resulting vertical displacement. (**B**) Top view deformation of the substrate under horizontal loading. The gray square represents the initial (unloaded) position of the substrate, while the transparent square indicates its deformed state under load. Due to asymmetrical deformation, the springs on the side closer to the direction of applied force deform differently from those on the opposite side. The deformed lengths of these springs are computed separately based on geometric constraints. Force analysis is then used to establish the relationship between horizontal load and horizontal displacement.

The vertical stiffness calculation model is illustrated in Figure 3A. Suppose the substrate is subjected to a vertical load $F_{Vertical}$, resulting in a vertical displacement Δx. Let the original length of the spring be $l_{0}$, and its length after deformation be $l_{1}$. Then, the following relationship holds:

(3) $$l_{1}=\sqrt{\Delta x^{2}+l_{0}^{2}}$$

In addition:

(4) $$\left\{\begin{array}{l} F_{Spring}=K(l_{1}-l_{0})\\ F_{Spring}\cos\theta =F_{Vertical}/4\\ \cos\theta =\Delta x/l_{1} \end{array}\right.$$

According to Equations (3) and (4):

(5) $$F_{Vertical}=4K\Delta x(1-\frac{l_{0}}{\sqrt{\Delta x^{2}+l_{0}^{2}}})$$

So,

(6) $$K_{Vertical}=F_{Vertical}/\Delta x=4K(1-\frac{l_{0}}{\sqrt{\Delta x^{2}+l_{0}^{2}}})$$

The horizontal stiffness calculation model is shown in Figure 3B. When a horizontal force is applied to the substrate, it induces a horizontal displacement Δy. Under this loading condition, the springs on the side closer to the direction of the applied force undergo compression, while those on the opposite side experience tension. Therefore, the geometric relationships for spring deformation must be calculated separately for the two cases. Let the original (unloaded) length of each spring be $l_{0}$. After loading, the length of the spring on the side closer to the applied force is $l_{1}$, and the length of the spring on the far side is $l_{2}$. The following relationships then hold:

(7) $$\left\{\begin{array}{l} l_{1}=\sqrt{\frac{l_{0}^{2}}{2}+{\left(\Delta y+\frac{l_{0}}{\sqrt{2}}\right)}^{2}}\\ l_{2}=\sqrt{\frac{l_{0}^{2}}{2}+{\left(\Delta y-\frac{l_{0}}{\sqrt{2}}\right)}^{2}} \end{array}\right.$$

Let the force exerted on the spring located on the side closer to the direction of loading be denoted as $F_{Spring(long)}$, and the force on the spring on the opposite side as $F_{Spring(short)}$. Then, the relationship between spring deformation and the corresponding forces can be expressed as follows:

(8) $$\left\{\begin{array}{l} F_{Spring\left(long\right)}=K(l_{1}-l_{0})\\ F_{Spring\left(short\right)}=K(l_{2}-l_{0})\\ \tan\theta_{1}=\frac{l_{0}}{\sqrt{2}\Delta y+l_{0}}\\ \tan\theta_{2}=\frac{l_{0}}{\sqrt{2}\Delta y-l_{0}}\\ 2F_{Spring\left(long\right)}\cos\theta_{1}+2F_{Spring\left(short\right)}\cos\theta_{2}=F_{Horizon} \end{array}\right.$$

The compliance characteristics of the substrate, as determined through the model calculations, are summarized in Table 1.

To ensure that the performance testing apparatus allowed us to accurately estimate the substrate reaction forces generated during tree frog jumps, its measurement accuracy was evaluated before experimentation. First, the original positions of the marker points were recorded using the multi-camera system. Then, a tree frog with a known body weight was gently placed at the center of the force measurement substrate. After the frog remained stationary and the substrate stabilized, the positions of the marker points were captured again using the camera system. Based on the previously described force calculation method, the average reaction force exerted on the substrate over a short period of time was computed. This procedure was repeated three times for each substrate configuration with different spring stiffness combinations. The vertical ground reaction forces measured in the three trials were averaged, and the standard deviation was calculated. Finally, the measured forces were normalized by the body weight of the frog to obtain unitized values. The summarized results are presented in Table 2.

Three high-speed cameras (Cameras 1–3; Blackfly, Teledyne FLIR, Wilsonville, OR, USA) with a frame rate of 425.17 FPS were used to synchronously record substrate motion, while Camera 4 (GoPro Hero 8; GoPro, Inc., San Mateo, CA, USA) recorded the jumping behavior of the tree frogs at 239.76 FPS. In the experiments, a soft sponge board (1 m × 0.6 m × 0.1 m), positioned 41 cm from the center of the takeoff substrate, was vertically fixed to the ground as the landing substrate to reduce impact forces and minimize the risk of injury during tree frog landings (Figure 2A). Preliminary tests revealed that tree frogs prefer to land on darker surfaces. Therefore, black paper of equal size was affixed to the sponge surface to guide the frogs’ landing behavior.

Each frog was subjected to a maximum of three trials every 2 days. The frogs were placed on the takeoff substrate and then encouraged to jump by slightly touching their anus with a writing brush. If a tree frog’s jump trajectory deviates from the camera’s tracking plane, the corresponding experimental data will be excluded from analysis. To ensure data reliability, each frog was required to complete at least seven valid jumps on each compliant substrate.

### 2.3. Analyses

Video Data Processing: The recorded videos were used to calculate the reaction forces of the takeoff substrate and to capture the complete jumping sequence of the tree frogs. Accurate tracking was achieved by placing markers at the four corners of the substrate, both ends of each spring, and near the frog’s COM. First, the motion trajectories of the marker points were tracked using the DLTdv8 (version 8.2.0) in MATLAB R2020a (MathWorks, Natick, MA, USA) [29]. A combination of automated and manual tracking was employed: when markers were occluded or located at the edge of the video frame, manual frame correction was applied. The processed trajectory data were then imported into MATLAB (R2020a, MathWorks, Natick, MA, USA) for kinematic and dynamic analysis.

Force estimation and processing based on marker trajectories: Before the experiments, the spatial coordinates of the marker points and the fixed ends of the springs were measured in the unloaded state of the substrate. These data were used to construct a physical model of the substrate in a global coordinate system. Camera calibration was also performed to establish the relationship between pixel coordinates and real-world distances. During substrate reaction force calculation, the 3D positions of the four marker points on the carbon fiber plate were determined based on their displacements from their initial positions. Using Equations (1) and (2), the resulting force vectors acting on the substrate were decomposed into fore–aft, normal, and lateral components (Figure 2B). The time window for substrate reaction force analysis was defined from the onset of marker movement to the moment the frog’s feet completely left the carbon fiber plate. The raw force data were processed using a Butterworth low-pass filter to eliminate high-frequency noise. The time-varying profile of the takeoff forces on compliant substrates is illustrated in Figure 2B. It is worth noting that although small peaks in lateral force were occasionally observed—likely due to asynchronous hindlimb extension or non-linear jumping trajectories—these were relatively minor compared to the fore–aft and normal forces. Therefore, lateral force peaks were not included in the subsequent peak force comparison analysis.

Kinematic Data Processing: In studies of tree frog jumping, the hip (or vent) is commonly used as a proxy for the COM [30,31]. Based on this approximation, we calculated takeoff velocity, takeoff duration, and takeoff angle; Takeoff duration was defined as the time interval from the initiation of movement to the complete detachment of the hindlimbs from the substrate; Takeoff velocity was calculated as the velocity of the COM at the moment when the frog’s toes lost contact with the substrate, calculated as the displacement between adjacent frames divided by the time interval; Takeoff angle was calculated as the angle between the linear fit of the COM trajectory over the last 10 frames of takeoff (approximately 27 ms) and the horizontal plane. Kinematic trajectories were smoothed using polynomial spline functions. To eliminate potential confounding effects of the landing substrate on jumping performance, only trials in which the frog landed above the substrate height were included in the final analysis. The push-off phase is defined as the period from the initiation of movement by the tree frog on the substrate to the moment when the hindlimb toes completely lose contact with the substrate. This process is further divided into two distinct phases: the loading phase and the unloading phase. The loading phase refers to the interval from the onset of movement to the point at which the substrate reaches its maximum deformation. The unloading phase spans from the moment of maximum substrate deformation to the instant the frog’s toes lift off the substrate.

To quantify the energy exchange between the tree frog and the substrate, we calculated the energy components of the system.

Total System Energy:

(9) $$E_{sys}=E_{frog}+E_{spring}+E_{substrate\;motion}$$

Here, $E_{sys}$ represents the total energy of the system; $E_{frog}$ is the sum of the tree frog’s kinetic energy and gravitational potential energy; $E_{spring}$ denotes the elastic potential energy stored in the springs; and $E_{substrate\;motion}$ represents the sum of the kinetic and gravitational potential energy of the substrate. The change in the mechanical energy of the tree frog can be calculated using the following equation:

(10) $$E_{frog}=\frac{1}{2}m_{frog}v_{frog}^{2}+m_{frog}gh_{frog}$$

Here, $m_{frog}$ is the mass of the tree frog, and $v_{frog}$ is the magnitude of the velocity in the direction of the COM motion. The takeoff velocity is defined as the instantaneous velocity measured in the first frame after the frog’s hindlimbs completely leave the ground. It is calculated as the displacement of the COM between the frame before and after takeoff divided by the time interval between those frames. g is the acceleration due to gravity, and h is the vertical displacement of the COM from the onset of motion to the moment the toes leave the substrate. The elastic potential energy stored in the substrate is given by:

(11) $$E_{spring}=\frac{1}{2}k{\left(\Delta x_{1}\right)}^{2}+\frac{1}{2}k{\left(\Delta x_{2}\right)}^{2}+\frac{1}{2}k{\left(\Delta x_{3}\right)}^{2}+\frac{1}{2}k{\left(\Delta x_{4}\right)}^{2}$$

Here, *k* is the spring stiffness, $\Delta x_{i}$ represents the deformation of the i = 1, 2, 3, 4, and there are four springs in total. The kinetic and potential energy of the moving substrate is given by:

(12) $$E_{substrate\;motion}=\frac{1}{2}m_{substrate}v_{substrate}^{2}+m_{substrate}gh_{substrate}$$

Here, $m_{substrate}$ is the mass of the substrate (14 g in this study), $v_{substrate}$ is the velocity of the substrate, g is the acceleration due to gravity, and $h_{substrate}$ is the vertical displacement of the diagonal intersection point formed by the four substrate markers, measured from the onset of motion to the moment when the frog’s toes leave the substrate.

The total energy of the substrate, $E_{substrate}$ is the sum of its elastic potential energy and motion-related mechanical energy:

(13) $$\eta =\frac{E_{substrate,\max}-E_{substrate,toeoff}}{E_{substrate,\max}}$$

Here, $E_{substrate,\max}$ represents the energy of the substrate at the point of maximum elastic deformation, and $E_{substrate,toeoff}$ denotes the substrate energy at the moment the frog’s toes leave the substrate.

At the onset of the jump, all energy values were initialized to zero. Power and energy were normalized by the mass of the frog and the substrate, forces were normalized by the frog’s body weight, and substrate displacement was normalized by the frog’s SVL. In the unloaded state of the substrate, the spatial positions of the four fluorescent markers attached to the corners of the carbon fiber plate and the fixed ends of the four springs were recorded to establish a physical model of the substrate in the global coordinate system. Before experimentation, the cameras were calibrated to determine the pixel-to-length ratio in the world coordinate system. The displacement of the substrate and the ground reaction forces during takeoff were calculated by tracking the positions of the four markers on the carbon fiber plate and computing their spatial displacements (Δx, Δy, Δz). The diagonal intersection point of the four markers was used to determine the overall movement of the substrate.

Statistical Analysis: A mixed-effects ANOVA model was used to compare the frogs’ performance across substrates with different compliance levels, while accounting for individual variation and jump sequence as random effects. Fixed effects included substrate compliance and trial order, and random effects accounted for individual differences among frogs. For indices with significant main effects, pairwise comparisons between groups were conducted using Bonferroni-corrected post hoc tests, with the significance level set at α = 0.05. All statistical analyses were performed using SPSS 26.0 software (IBM, Armonk, NY, USA).

## 3. Results

### 3.1. Substrate Parameter Analysis

**Figure 4:**
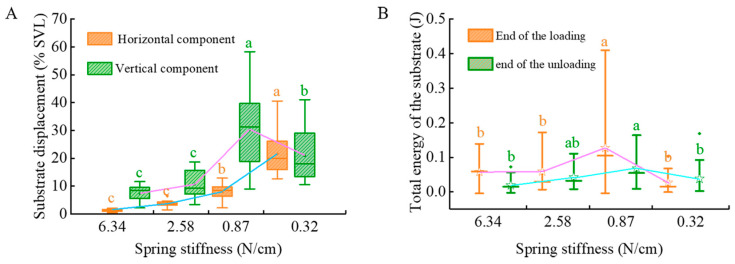
Effects of substrate compliance on substrate motion. (**A**) Substrate displacement; (**B**) Total substrate energy (sum of kinetic and potential energy). Spring stiffness is shown in descending order, with sample sizes of 37, 17, 28, and 18, respectively. Significant comparisons are indicated with a, b, ab and c; means not sharing the same letters are significantly different (*p* < 0.05).

During the push-off phase, the substrate, constrained by springs, exhibited both vertical and horizontal displacements. The vertical displacement component, generated by the vertical substrate reaction force from the limbs, ranged from 2% to 58% of the SVL, as shown in Figure 4A. Mixed-effects model analysis revealed a significant main effect of substrate compliance on vertical displacement during takeoff (*F* _(3,92)_ = 12.895, *p* < 0.001). The substrate with spring stiffness of 0.32 N/cm produced significantly greater vertical displacement (19.47 ± 6.13% SVL, *p* = 0.002) than that with spring stiffness of 6.34 N/cm. Notably, the substrate with spring stiffness of 0.87 N/cm yielded the greatest vertical displacement (27.74 ± 4.75% SVL), which was significantly greater than that at all other stiffness levels (*p* ≤ 0.007). The horizontal displacement component, caused by the backward propulsive force, ranged from 0.43% to 40.57% SVL. Statistical analysis showed that substrate compliance had a highly significant effect on horizontal displacement during takeoff (*F*_(3,92)_ = 35.019, *p* < 0.001). Post hoc comparisons revealed significant differences among spring stiffness conditions. The substrate with spring stiffness of 0.32 N/cm exhibited the greatest horizontal displacement (23.15 ± 2.32% SVL, *p* < 0.001), followed by those with stiffness values of 0.87 N/cm (8.03 ± 1.80% SVL) and 2.58 N/cm (2.48 ± 2.30% SVL), while the substrate with 6.34 N/cm yielded the least displacement.

The mechanical energy of the substrate is shown in Figure 4B. At the end of the loading phase, the substrate with a spring stiffness of 0.87 N/cm exhibited significantly higher mechanical energy (0.123 ± 0.013 J) compared to the substrate with spring stiffness of 0.32 N/cm (0.025 ± 0.015 J, *p* < 0.001), 2.58 N/cm (0.059 ± 0.016 J, *p* = 0.015) and 6.34 N/cm (0.058 ± 0.011 J, *p* = 0.001). No significant difference was found among the substrate with spring stiffnesses of 0.32 N/cm, 2.58 N/cm and 6.34 N/cm (all *p* > 0.05). During the unloading phase, the substrate with spring stiffness of 0.87 N/cm again exhibited the highest mechanical energy (0.067 ± 0.007 J), which was significantly greater than that of the substrates with spring stiffness of 0.32 N/cm (0.038 ± 0.008 J, *p* = 0.040) and 6.34 N/cm (0.019 ± 0.006 J, *p* < 0.001). The substrate with spring stiffness 2.58 N/cm (0.043 ± 0.008 J) did not differ significantly from the other conditions (*p* > 0.05).

### 3.2. Kinematic Parameter Analysis of Tree Frogs

**Figure 5:**
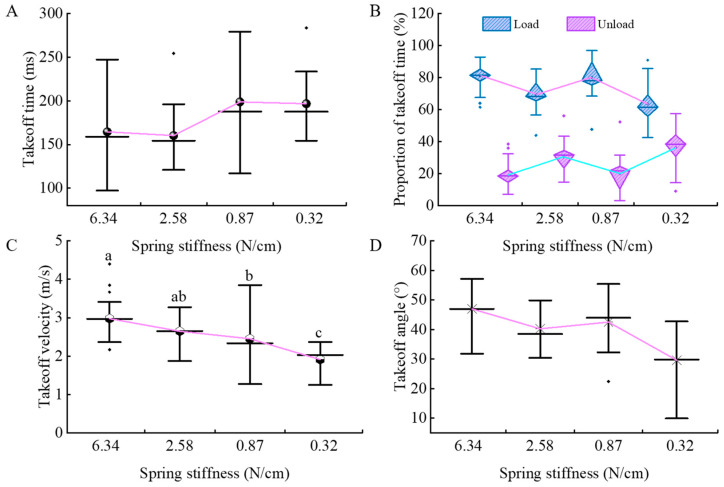
Effect of substrate compliance on the jumping kinematics of tree frogs. (**A**) Takeoff time; (**B**) Proportion of loading and unloading phases relative to the total takeoff time; (**C**) Takeoff velocity and (**D**) Takeoff angle. Spring stiffness is shown in descending order, with sample sizes of 37, 17, 28, and 18, respectively. Significant comparisons are indicated with a, b, ab and c; means not sharing the same letters are significantly different (*p* < 0.05).

Mixed-effects model analysis showed that substrate compliance had no significant effect on multiple kinematic parameters of tree frogs’ jumping, specifically including: takeoff time (*F*_(3,92)_ = 0.485, *p* = 0.694, Figure 5A), the ratio of loading time to unloading time (*F*_(3,92)_ = 1.025, *p* = 0.385, Figure 5B), and takeoff angle (*F*_(3,92)_ = 1.901, *p* = 0.135, Figure 5D).

Only takeoff velocity was significantly affected by the main effect (*F*_(3,92)_ = 3.113, *p* = 0.030, Figure 5C). Bonferroni-corrected post hoc comparisons revealed that the takeoff velocity on the 0.32 N/cm substrate (1.908 ± 0.126 m/s) was significantly lower than those on the other substrates: 0.87 N/cm (2.452 ± 0.101 m, *p* = 0.007), 2.58 N/cm (2.649 ± 0.130 m/s, *p* = 0.001), and 6.34 N/cm (2.976 ± 0.088 m/s, *p* < 0.001). Additionally, takeoff velocity on the 0.87 N/cm substrate was significantly lower than that on the 6.34 N/cm substrate (*p* = 0.001). These results indicate that as the substrate compliance increases, the takeoff speed decreases.

### 3.3. Dynamic Parameter Analysis of Tree Frogs

**Figure 6:**
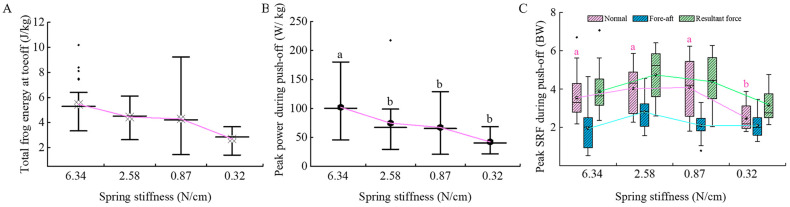
Dynamic parameters during takeoff in tree frogs. (**A**) Takeoff mechanical energy; (**B**) Peak power during push-off and (**C**) Peak SRF during push-off, SRF stands for Substrate Reaction Force. Spring stiffness is shown in descending order, with sample sizes of 37, 17, 28, and 18, respectively. Significant comparisons are indicated with a and b; means not sharing the same letters are significantly different (*p* < 0.05).

Mixed-effects model analysis revealed that substrate compliance had no significant effect on the total mechanical energy of tree frogs’ jumps (*F*_(3,92)_ = 2.488, *p* = 0.065, Figure 6A), but had a significant effect on their peak power during push-off (*F*_(3,92)_ = 3.851, *p* = 0.012 Figure 6B). Compared to the 6.34 N/cm substrate, the 0.32 N/cm substrate led to a significant reduction in jump power (*p* = 0.002), and a similar reduction was observed with the 0.87 N/cm substrate (*p* = 0.020).

During push-off, tree frogs generate substrate reaction forces up to 6 times their body weight in the normal direction and approximately 4.5 times in the fore-aft direction, as illustrated in Figure 6C. Statistical analysis revealed that substrate compliance had a significant main effect on peak normal force (*F*_(3,92_) = 2.923, *p* = 0.038). Bonferroni-corrected post hoc comparisons showed that the 0.32 N/cm substrate produced a significantly lower peak normal force (2.485 ± 0.259 BW) than the other substrates, including 0.87 N/cm (4.188 ± 0.214 BW, *p* < 0.001), 2.58 N/cm (4.030 ± 0.275 BW, *p* = 0.001) and 6.34 N/cm (3.554 ± 0.186 BW, *p* = 0.007). However, substrate compliance had no significant main effect on either peak fore-aft force (*F*_(3,92)_ = 2.255, *p* = 0.087) or peak resultant force (*F*_(3,92)_ = 1.625, *p* = 0.189).

## 4. Discussion

We investigated how the compliance of the horizontal launching substrate affects the jumping performance of tree frogs when landing on vertical substrate. Six types of commercially available springs with stiffness values of 6.34, 2.58, 0.87, 0.32, 0.11, and 0.03 N/cm were selected, with each adjacent pair demonstrating an approximately threefold difference in stiffness. Our preliminary experiments revealed a critical threshold: when spring stiffness was ≤0.11 N/cm, tree frogs were unable to execute successful jumps. Although the frogs initiated takeoff movements, they consistently failed to detach their toe pads from the substrate. This phenomenon resulted from excessive substrate compliance, which prevented the generation of sufficient propulsive force for complete detachment. A similar failure pattern has also been reported in click beetles (Elateridae) when attempting to jump on overly compliant substrates [14], suggesting a common biomechanical constraint among jumping animals.

Across successful takeoffs (stiffness ≥ 0.32 N/cm), substrate compliance clearly influenced both frog movements and substrate dynamics, yet only horizontal substrate displacement and frog takeoff velocity exhibited statistically significant changes across the full range of compliant substrates. Notably, the trend in takeoff velocity aligns with previous findings [28], highlighting the general effect of substrate compliance on amphibian jumping performance. While total mechanical energy and peak power at toe detachment showed a downward trend with increasing compliance, these changes were not statistically significant.

**Figure 7:**
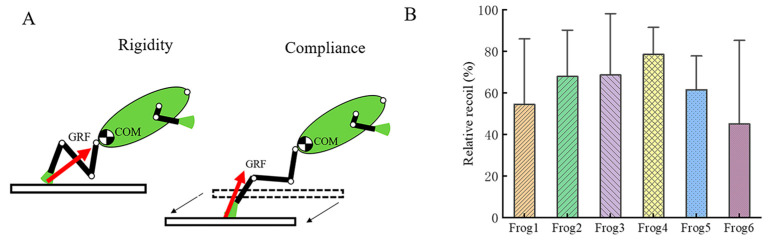
(**A**) Mechanical model of the effect of substrate compliance on takeoff angle and (**B**) Energy efficiency. The number of jumps for each tree frog was 6, 5, 6, 6, 6, and 7 respectively.

Although takeoff angle was not statistically significant, the takeoff angle on the 0.32 N/cm substrate was smaller than that observed on the 6.34 N/cm substrate. Mechanical modeling suggests that on rigid substrates, the direction of the substrate reaction force is consistently aligned with the COM, minimizing torque around the COM. However, on compliant substrates, deformation causes the substrate contact point to shift posteriorly, displacing the substrate reaction force vector behind the COM. This displacement generates a moment arm and induces a net clockwise torque, thereby reducing the inclination of the body’s longitudinal axis (Figure 7A). The greater the compliance, the more pronounced this mechanical effect becomes. Also, takeoff time was not statistically significantly affected by substrate compliance, the takeoff time on the 0.32 N/cm substrate was longer than that on the 0.87 N/cm substrate. A plausible explanation for this is that the flexible substrate disrupts the inertial capture mechanism through which tree frogs store elastic energy in their tendons, thereby increasing the duration of takeoff [27].

A distinct turning point was observed at the substrate stiffness of 0.87 N/cm. Parameters including vertical substrate displacement, total energy, as well as the frog’s takeoff time, takeoff angle, and takeoff force all exhibited this shift. A plausible explanation is that, in this experiment, 0.87 N/cm may represent a critical biomechanical boundary in the interaction between the tree frog’s locomotor system and the mechanical properties of the substrate. From an energy transfer perspective, substrates stiffer than 0.87 N/cm can provide sufficient rigidity to minimize energy loss due to excessive deformation, while still maintaining enough compliance to avoid harmful rebound forces.

Our study supports the findings of Astley et al. [28] that tree frogs performing static jumps on compliant substrates can recover a small portion of the energy stored in the substrate, as illustrated in Figure 7B. On a substrate with a spring stiffness of 6.34 N/cm, tree frogs recovered up to 50% of the stored elastic energy during takeoff. However, this energy recovery efficiency exhibited a pronounced dependence on substrate compliance and was only observed at the 6.34 N/cm spring stiffness level. As substrate compliance increased, the static loading caused by the frog’s body weight led to a significant initial deformation of the substrate, substantially reducing the amount of energy that could be stored during the loading phase. During the unloading phase, the substrate’s rebound exceeded the equilibrium position, resulting in dynamic overshoot that disrupted the energy balance. This nonlinear dynamic response indicates that traditional efficiency calculations based on energy conservation are no longer applicable under conditions of very high substrate compliance. When spring stiffness decreased to 0.11 N/cm, although the frogs could still perform the takeoff motion, the substrate was so compliant that their adhesive pads failed to fully detach from the surface. This suggests that the reactive forces generated by the substrate were insufficient to overcome gravity and fully propel the body into the air.

Force analysis further revealed that tree frogs can generate peak normal reaction forces up to 6 times their body weight and fore-aft forces of approximately 4.5 times their body weight during takeoff. This indicates that their robust musculoskeletal system has evolved to produce exceptionally high force relative to body mass, ensuring sufficient propulsion even when taking off from complex substrates. Moreover, the coordinated functions of the normal reaction force (to overcome gravity and initiate take-off) and the fore–aft force (to provide horizontal thrust and assist acceleration) enable the effective production of powerful jumps, precisely supporting their survival demands for crossing branch gaps and evading predator. Our further analysis revealed that substrate compliance had a significant effect on peak normal force, with the value at a substrate stiffness of 0.32 N/cm being significantly lower than those at all stiffer substrates. However, this reduction did not follow a progressive monotonic decrease with increasing compliance. Instead, the peak normal force was observed at a substrate stiffness of 0.87 N/cm. A possible explanation is that this intermediate stiffness provides an optimal balance between force transmission efficiency and substrate feedback, allowing the frogs to effectively exert vertical force and generate greater substrate reaction forces.

These findings offer new biomechanical insights into how adhesive jumping animals interact with compliant environments. The frogs are able to recover part of the energy from the compliant substrate, and their superior behavioral performance on substrates with intermediate to high stiffness, as indicated by higher take-off velocities, may represent an adaptive strategy that facilitates reliable detachment and effective propulsion. This helps explain the evolutionary preference of many climbing and jumping animals for stiffer substrates during locomotion [16,32,33,34]. Moreover, the principles uncovered in this study could be extended to the design of bioinspired systems, particularly in the context of material origami technologies. Recent advancements in bioinspired origami structures, such as origami springs and dual-morphing stretchable materials, highlight the potential for substrates with variable compliance to enhance energy efficiency and adaptive response in engineered systems [35,36]. These materials can be designed to mimic the compliance behavior observed in tree frogs, enabling the creation of multifunctional systems that respond dynamically to external forces. Furthermore, this research could play a key role in the miniaturization of bioinspired mechanical mechanisms, particularly for microrobot applications. As demonstrated in recent work on soft robotics, the interaction between compliant surfaces and adhesive mechanisms can be critical for achieving efficient locomotion and energy recovery in small scale systems [37]. By leveraging insights from tree frog locomotion, future bio-inspired microrobots could be designed to optimize force generation, energy transfer, and adaptability in complex, variable environments. These considerations open exciting avenues for the development of advanced bio-electronic systems and soft robotics, where material compliance plays a crucial role in both performance and efficiency.

## 5. Conclusions

Tree frogs demonstrate the ability to make behavioral decisions based on varying environmental conditions. This study systematically investigated the jumping dynamics and kinematics of frogs (*Polypedates dennysi*) on six substrate compliances to clarify the biomechanical mechanisms underlying their adaptation to variable arboreal substrates. The findings reveal that substrate compliance influences energy recovery during takeoff; on the stiffest substrates, tree frogs recovered up to 50% of the energy stored in the substrate, reflecting efficient energy utilization, while highly compliant substrates induced nonlinear damping, substantial energy dissipation, and even takeoff failure, indicating a critical threshold beyond which effective locomotion is compromised. Distinct adaptive patterns were observed between force generation and takeoff velocity. During takeoff, frogs produced peak normal forces up to six times their body weight and fore–aft forces up to 4.5 times; however, these force outputs exhibited limited adaptability to substrate compliance. By contrast, takeoff velocity showed greater resilience to compliance variation, suggesting a strategy that prioritizes maintaining movement efficiency over force regulation under substrate variability. Collectively, these results reveal a trade-off between substrate mechanics and jump performance, underscoring the evolutionary sensitivity of jumping animals to the physical properties of their environment. Furthermore, these findings provide valuable biomechanical insights for the development of bioinspired systems capable of efficient locomotion under non-ideal conditions.

## Figures and Tables

**Table 1:** Compliance calculation results of the substrate.

Spring Stiffness (N/cm)	Horizontal Stiffness (N/cm)	Vertical Stiffness (N/cm)
6.34	11.41	2.33
2.58	4.64	0.96
0.87	1.56	0.37
0.32	0.57	0.18
0.11	0.19	0.11
0.03	0.04	0.08

**Table 2:** Measurement accuracy of substrate reaction forces.

Spring Stiffness (N/cm)	Load (BW)	Measurement Result (BW)	Relative Error
6.34	1	0.89 ± 0.02	11%
2.58	1	0.96 ± 0.04	4%
0.87	1	0.91 ± 0.01	9%
0.32	1	0.87 ± 0.01	13%

Note: BW represents the body weight of the tree frog. The measurement results are presented as the mean ± standard deviation of three repeated trials.

## Data Availability

The data generated and/or analyzed in the current study are not publicly available for legal/ethical reasons but are available from the corresponding author upon reasonable request.

## Abbreviations

The following abbreviations are used in this manuscript:

COM	Center of Mass
SVL	Snout–Vent Length
BW	Body Weight
